# miRNA-Based Feature Classifier Is Associated with Tumor Mutational Burden in Head and Neck Squamous Cell Carcinoma

**DOI:** 10.1155/2020/1686480

**Published:** 2020-12-09

**Authors:** Yu Xia, Qi Wang, Xiaolin Huang, Xinhai Yin, Jukun Song, Zhao Ke, Xiaofeng Duan

**Affiliations:** ^1^Department of Oral Medicine, Guizhou Provincial People's Hospital, Guizhou, China; ^2^Department of Orthodontics, Guizhou Provincial People's Hospital, Guizhou, China; ^3^School of Stomatology, Guizhou Medical University, Guiyang, Guizhou, China; ^4^Department of Oral and Maxillofacial Surgery, Guizhou Provincial People's Hospital, Guizhou, China

## Abstract

Tumor mutation burden (TMB) is considered to be an independent genetic biomarker that can predict the tumor patient's response to immune checkpoint inhibitors (ICIs). Meanwhile, microRNA (miRNA) plays a key role in regulating the anticancer immune response. However, the correlation between miRNA expression patterns and TMB is not elucidated in HNSCC. In the HNSCC cohort of the TCGA dataset, miRNAs that were differentially expressed in high TMB and low TMB samples were screened. The least absolute contraction and selection operator (LASSO) method is used to construct a miRNA-based feature classifier to predict the TMB level in the training set. The test set is used to verify the classifier. The correlation between the miRNA-based classifier index and the expression of three immune checkpoints (PD1, PDL1, and CTLA4) was explored. We further perform functional enrichment analysis on the miRNA contained in the miRNA-based feature classifier. Twenty-five differentially expressed miRNAs are used to build miRNA-based feature classifiers to predict TMB levels. The accuracy of the 25-miRNA-based signature classifier is 0.822 in the training set, 0.702 in the test set, and 0.774 in the total set. The miRNA-based feature classifier index showed a low correlation with PD1 and PDL1, but no correlation with CTLA4. The enrichment analysis of these 25 miRNAs shows that they are involved in many immune-related biological processes and cancer-related pathways. The miRNA expression patterns are related to tumor mutation burden, and miRNA-based feature classifiers can be used as biomarkers to predict TMB levels in HNSCC.

## 1. Introduction

Head and neck squamous cell carcinoma (HNSCC) remains the primary cause of cancer-related mortality in the world, which is characterized by advanced diagnosis [1], poor prognosis [2], low overall survival [3], and high recurrence [4]. HNSCC is regarded as the tenth most common cancer in the world and the seventh most common cause of cancer deaths. There are approximately 400,000 oral and pharyngeal diseases, 160,000 laryngeal cancers, and 300,000 deaths worldwide each year [5–7]. HNSCC is a common heterogeneous tumor that exists in the oral, pharyngeal, and larynx lesions [8]. Risk factors for oral cancer may include mutant oncogenes, the presence of extensive P53, and lower levels of tumor hypoxia, smoke, alcohol, age factor (>65 years), and HPV or EBV infection, but the mechanism remains to be explored in detail. Existing treatments are deficient for patients with locally advanced or distantly metastatic HNSCC. Therefore, looking for a new treatment method is currently urgent.

At present, tumor immunotherapy has been the main driving force for personalized precision medicine, and efforts are being made to use the immune system to treat advanced or metastatic cancers [9–12]. The main categories of immunotherapy include monoclonal antibody immune checkpoint inhibitors (ICIs), therapeutic antibodies, cancer vaccines, immune system regulators, cell therapy, and synthetic small molecule inhibitors. In recent years, the most worth mentioning is that HNSCC has made a breakthrough in immunotherapy, especially in the discovery of inhibitors of the immune pathway-related checkpoint, such as antiprogrammed death 1 (anti-PD1) and anti-CTLA4, which have important effects on the treatment of locally advanced metastatic HNSCC [13–15].

Tumor mutation load (TMB) is a new type of biomarker that predicts the effect of immunotherapy. Multiple studies have investigated the prognostic role of TMB in immunotherapy for multiple cancer types [16–18]. The translation of mutated genes into modified proteins requires posttranscriptional regulation, and microRNA (miRNA) is an important molecule involved in posttranscriptional regulation. Meanwhile, the abnormal expression of miRNAs was involved in the occurrence of cancer, and it has attracted people's attention. Previous studies have shown that miRNA may act as a prognostic indicator in many types of cancers [19, 20]. Recently, people are paying more and more attention to the role of miRNA in regulating the anticancer immune response [21, 22]. It has been clarified that miRNA is involved in mediating and controlling various immune and cancer cell interactions. Therefore, we hypothesized that miRNA expression patterns can be used as biomarkers to predict TMB levels. To verify our hypothesis, we downloaded the data of the head and neck squamous cell carcinoma (HNSCC) dataset from TCGA, including mutation annotation files and miRNA expression profiles, and constructed miRNA-based feature classifiers to predict TMB levels.

## 2. Materials and Methods

### 2.1. Data Acquisition and Processing

First, the mRNA and miRNA profiling was downloaded from the TCGA database by the GDC tool (https://portal.gdc.cancer.gov/ ), including 502 HNSCC patients and 44 adjacent-tumor samples. Then, we excluded the adjacent-tumor samples and retained the tumor samples. We also obtained the somatic mutation data of all patients in the “Masked Somatic Mutation” category of TCGA processed by VarScan software. Besides, clinical information of each patient, including age, gender, TNM stage, tumor grade, and survival time, is obtained through the GDC portal.

**Figure 1:**
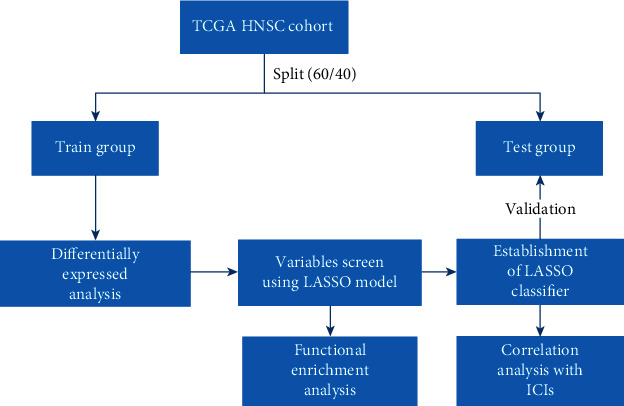
Flowchart delineates the study design and analysis process.

In the end, a total of 501 samples with both miRNA expression and mutation profiling were considered eligible in this analysis. These samples were randomly assigned to the training (60%) and test (40%) sets. The flowchart of this study is exhibited in Figure 1.

### 2.2. Assessment of TMB for Each Patient and Prognostic Analysis

Tumor mutational burden was defined as the number of somatic variants per megabase (MB) of the genome [23]. We employed the Kaplan-Meier analysis with the log-rank test to screen optimal TMB value. Then, the patients were divided into the high and low TMB groups according to the optimal cut-off point of the TMB value. Meanwhile, the TMB levels from the TCGA cohort were merged with corresponding survival data of each sample via merge function in R. Also, we further assess the relationship between the TMB groups with several clinical variables. We used the Wilcoxon rank-sum test to compare the clinical characteristics of the two groups, while the Kruskal-Wallis test is used for comparison between three or more groups.

### 2.3. Mutation Analysis

The mutation data in the MAF of HNSCC patients were used in the TCGA dataset. The R package “maftools” was used to display the mutation profile of each group [24]. The maftools was also used to impute the mutation rate of each gene and also identified significant mutant genes in different subtypes (*p* < 0.05).

### 2.4. Identification of Differentially Expressed miRNAs

The differentially expressed miRNAs between high TMB samples and low TMB samples were conducted using the “limma” package in R [25]. The differentially expressed miRNA of datasets with ∣log2 fold change | ≥1.0 and a *p* value less than 0.01 was considered the selection criteria for subsequent analysis. The heatmap plot was also exhibited.

### 2.5. Principal Component Analysis (PCA) and Least Absolute Shrinkage and Selection Operator (LASSO) Analysis

The expression values of differentially expressed miRNAs for each HNSCC sample were extracted in the training set. The LASSO logistic regression model was conducted using the “glmnet” package in R, which has a powerful predictive value and a low correlation between each other to prevent overfitting and applied to select optimal features for the high-dimensional data [26]. Before using the expression profiles of all the differently expressed miRNAs for feature selection, we first performed PCA to examine the distribution of samples. PCA was then performed using the expression profile of the best differentially expressed miRNA. The PCA plot was drawn across the first two principal components.

### 2.6. miRNA-Based Feature Classifier for Predicting TMB Level

We used the LASSO method to build a prediction model, selected nonzero regression coefficients to identify the optimal miRNA set, and used the selected miRNA to build a miRNA-based feature classifier to predict TMB levels. The regression coefficients from the LASSO analysis were used to create a classification index for each sample, and the following formula was used to weigh the expression value of the selected miRNA: The Prognostic Index = (Exp miRNA1∗Coef1) + (Exp miRNA2∗Coef2) + (Exp miRNA3∗Coef3) + ⋯+(Exp miRNAn∗Coefn). The test set is used to verify the robustness and portability of the classifier. The prognostic performance of the classifier was evaluated using receiver operating characteristic (ROC) curves by comparing the sensitivity and specificity of the survival prediction. The ROC curves were plotted using the “pROC” package in R [27].

### 2.7. Association between the miRNA-Based Signature Classifier Model and the Expression of Three Immune Checkpoints

The correlation between the miRNA-based signature classifier index and the expression of three immune checkpoints (PD1, PDL1, and CTLA4) was analyzed using the Spearman rank correlation analysis. A *p* value of less than 0.05 was considered statistically significant.

### 2.8. Identification of Target Genes and Functional Enrichment Analysis

starBase [28], TargetScan [29], and miRDB [30] were used to check whether these three immune checkpoints are target genes of any of these miRNAs. Besides, DIANA mirPath web server was used to perform the KEGG pathway and Gene Ontology (GO) enrichment analyses for these miRNAs [31]. The “ggplot2” package was used to visualize the enrichment results. The enrichment analysis was based on the threshold of *p* value < 0.05.

### 2.9. Correlation between miRNA-Based Signature Classifier and Pathways

Firstly, the gene set of the interested pathway was downloaded from the KEGG (Kyoto Encyclopedia of Genes and Genomes) dataset (http://www.genome.jp/kegg/). Secondly, single-sample gene set enrichment analysis (ssGSEA) was used to calculate the enrichment scores of the pathway. Thirdly, the relationship between miRNA-based signature classifier and pathways using the Spearman rank correlation analysis was studied.

## 3. Results

### 3.1. Comparisons of Clinical Feature between High and Low TMB Groups

**Figure 2:**
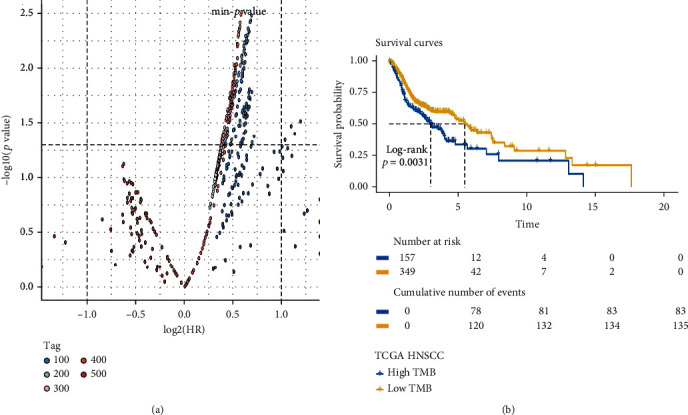
The Kaplan-Meier analysis was conducted to screen the optimal cut-off point. (a) The distribution of the *p* value and hazard ratio in all groups, the *p* value at the position marked by the horizontal dotted line is 0.05, and the HRs marked by the two vertical dotted lines are 0.5 and 2. (b) Survival curve of the best group.

**Figure 3:**
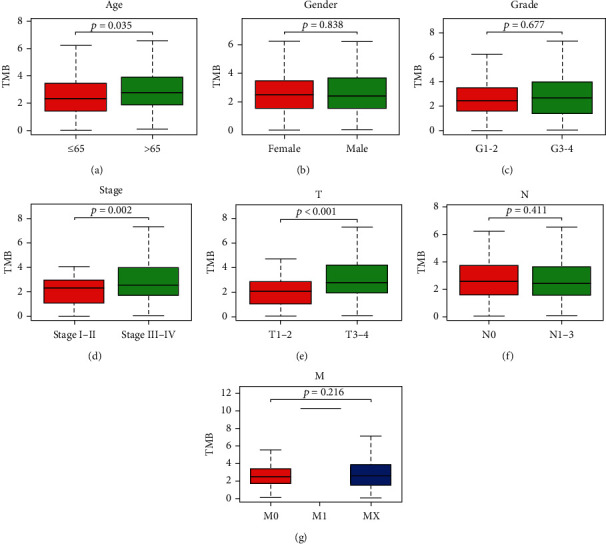
The clinical difference in age (a), gender (b), grade (c), TNM stage (d), T stage (e), N stage (f), and M stage (g) between the high TMB and low TMB groups.

We conducted the Kaplan-Meier analysis to screen the optimal cut-off point (TMB: 3.236842105); the survival plot is shown in Figures 2(a) and 2(b). Based on the optimal cut-off point, the HNSCC patients were divided into two groups: the high TMB and low TMB groups. We compared the clinical difference between the high and low TMB groups. The results indicated that the difference in TNM stage, age, and T stage between the high TMB and low TMB groups is observed in Figures 3(a)–3(g).

### 3.2. Comparisons of Gene Mutation between High and Low TMB Groups

**Figure 4:**
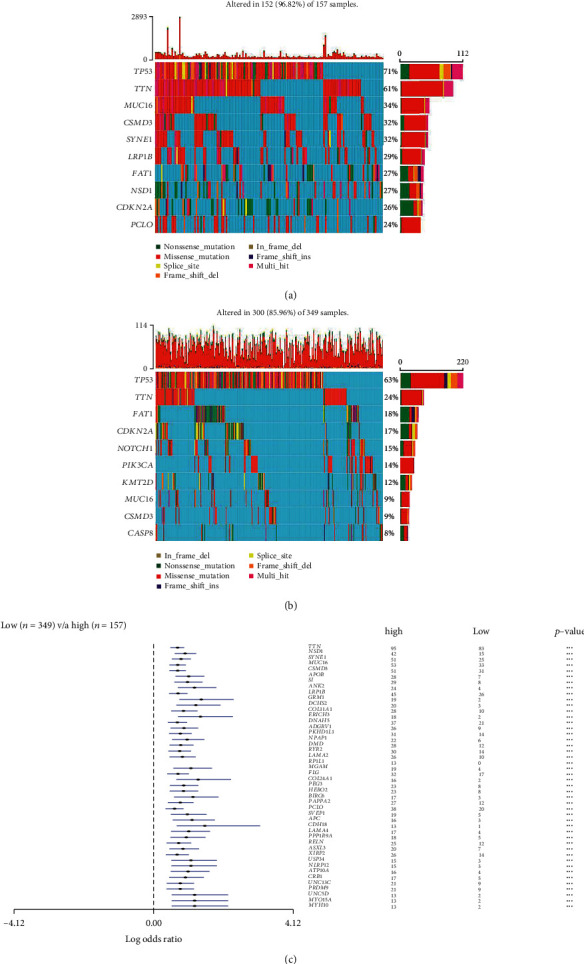
Mutation analysis between the high TMB and low TMB groups in the TCGA dataset. (a, b) Gene mutation profiles of the following highly mutated genes among the two subtypes. (c) The forest plots show the comparison results of gene mutations (^∗^*p* = 0.1, ^∗∗^*p* = 0.05, ns: not significant).

This study examined the association between the high and low TMB groups and the count of somatic mutation. Gene mutation profiles of these highly mutated genes are shown in Figures 4(a) and 4(b). In the high TMB group, TP53, TTN, and MUC16 were the most mutated genes, while TP53, TTN, and FAT1 were the most mutated genes in the low TMB group. MUC16 showed a higher mutation rate in the high TMB group, and FAT1 exhibited a higher mutation rate in the low TMB group with the cut-off point less than 0.05 (Figure 4(c)).

### 3.3. The Screen of Differentially Expressed miRNAs

**Figure 5:**
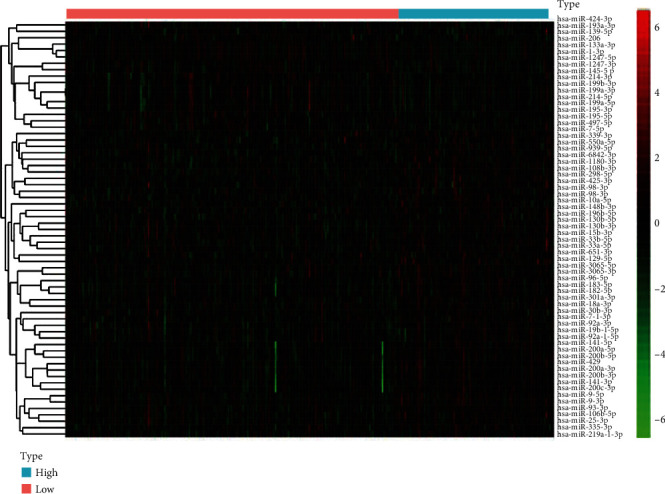
The heatmap plot of differentially expressed miRNAs between the high TMB and low TMB groups was exhibited.

In both the train and test groups, there are no statistically significant disparities in basic clinical features in Table 1. The 206 samples with a high TMB level and 95 samples with a low TMB level were included in the training group. We conducted differentially expressed analysis in the training group that a total of 65 differentially expressed miRNAs, including 55 upregulated miRNAs and 10 downregulated miRNAs, were identified with the threshold of cut-off point (*p* < 0.01 and ∣log2FC | >1.0). The heatmap plot of differentially expressed miRNAs is exhibited in Figure 5.

### 3.4. Feature Selection Using the LASSO Model

**Figure 6:**
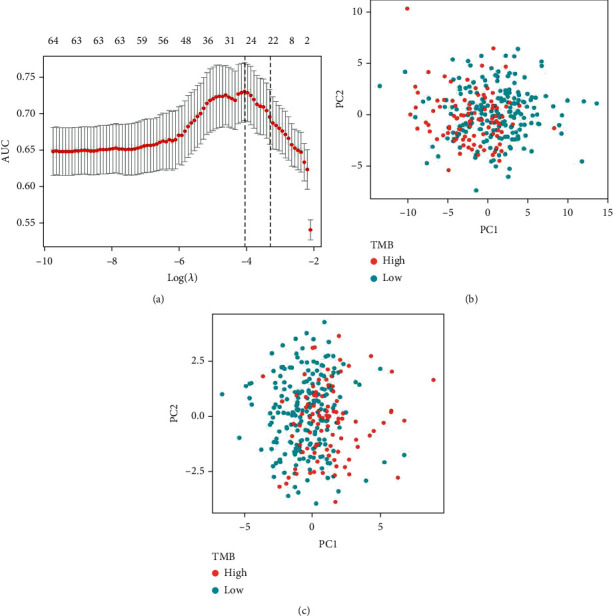
LASSO regression model and PCA. (a) 10-fold cross-validation for adjusting the parameter selection in the LASSO model. PCA (b) before and (c) after LASSO variable reduction.

In order to establish a miRNA-based feature classifier for prediction of the TMB level in HNSCC, we performed the LASSO logistic regression method to screen crucial miRNAs in the training set. We calculated the 10-fold cross-validation and type grouping classification. Measure = “auc” is used for two types of logistic regression to obtain the AUC curve. In the LASSO logistic regression method, twenty-five miRNAs as optimal features were ultimately recognized, including hsa-miR-195-5p, hsa-miR-141-5p, hsa-miR-195-3p, hsa-miR-10a-5p, hsa-miR-33b-5p, hsa-miR-98-5p, hsa-miR-6842-3p, hsa-miR-424-3p, hsa-miR-339-3p, hsa-miR-7-5p, hsa-miR-19b-1-5p, hsa-miR-296-5p, hsa-miR-939-5p, hsa-miR-139-5p, hsa-miR-96-5p, hsa-miR-9-3p, hsa-miR-3065-3p, hsa-miR-301a-3p, hsa-miR-106b-3p, hsa-miR-497-5p, hsa-miR-98-3p, hsa-miR-425-3p, hsa-miR-1247-5p, hsa-miR-18a-3p, and hsa-miR-193a-3p (Figure 6(a)). We also performed principal component analysis prior to and after the LASSO method, and the results indicated that samples with different TMB levels are more easily distinguished using the 25 miRNAs (Figures 6(b) and 6(c)).

**Figure 7:**
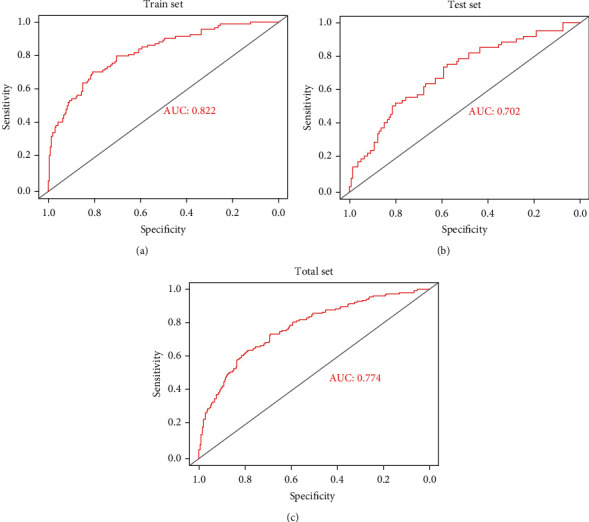
Receiver operating characteristic curves for the 25-miRNA-based signature model. (a–c) The train, test, and total sets, respectively.

We used the LASSO method to make a 10-fold cross-validation and identified 25 miRNAs with nonzero regression coefficients, and the value of lambda.min = 0.01222832. The miRNA-based classifier index was calculated using the following formula: hsa‐miR‐195‐5p∗(−0.08518) + hsa‐miR‐141‐5p∗0.112407 + hsa‐miR‐195‐3p∗(−0.1567) + hsa‐miR‐10a‐5p∗0.078228 + hsa‐miR‐33b‐5p∗0.16082 + hsa‐miR‐98‐5p∗0.097853 + hsa‐miR‐6842‐3p∗0.190134 + hsa‐miR‐424‐3p∗(−0.22954) + hsa‐miR‐339‐3p∗0.041014 + hsa‐miR‐7‐5p∗0.139587 + hsa‐miR‐19b‐1‐5p∗0.025402 + hsa‐miR‐296‐5p∗0.116113 + hsa‐miR‐939‐5p∗0.117358 + hsa‐miR‐139‐5p∗(‐0.10164) + hsa‐miR‐96‐5p∗0.063381 + hsa‐miR‐9‐3p∗0.129871 + hsa‐miR‐3065‐3p∗0.046985 + hsa‐miR‐301a‐3p∗0.114802 + hsa‐miR‐106b‐3p∗0.003671 + hsa‐miR‐497‐5p∗(‐0.45308) + hsa‐miR‐98‐3p∗0.452075 + hsa‐miR‐425‐3p∗0.121309 + hsa‐miR‐1247‐5p∗(‐0.0321) + hsa‐miR‐18a‐3p∗0.114407 + hsa‐miR‐193a‐3p∗(‐0.30399). The accuracy of the 25-miRNA-based classifier was 0.822 in the training set, 0.702 in the test set, and 0.774 in the total set (Figures 7(a)–7(c) and Table 2).

### 3.5. Correlation between the LASSO Classifier Index and Three Immune Checkpoint Inhibitors

**Figure 8:**
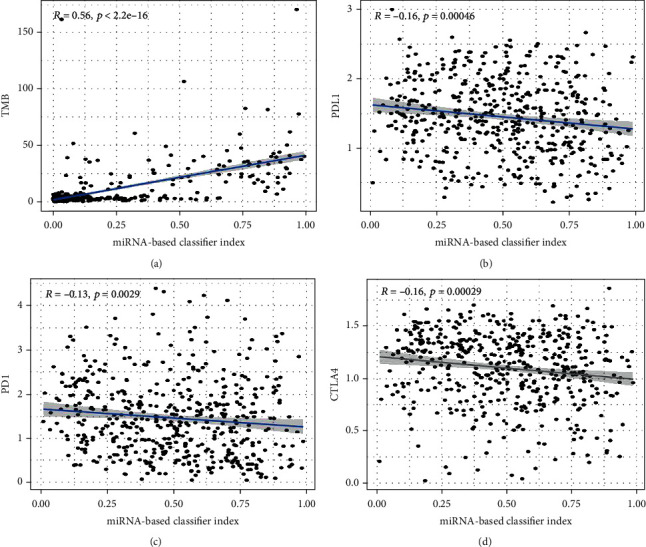
The correlation between the miRNA-based classifier index and TMB (a), PDL1 (b), PD1 (c), and CTLA4 (d).

We calculate the classification index of all samples based on the classifier and calculate the correlation of the classification index with TMB and the expression of three immune checkpoints (PD1, PDL1, and CTLA4) in the total set. Meanwhile, as a classifier for predicting TMB, the miRNA-based classifier index is highly correlated with TMB (Pearson *R* = 0.56, *p* < 0.001) (Figure 8(a)). The miRNA-based classifier index showed a low correlation with PD1 (Pearson *R* = −0.13, *p* = 0.0029, Figure 8(b)), PDL1 (Pearson *R* = −0.16, *p* = 0.00046, Figure 8(c)), and CTLA4 (Pearson *R* = −0.16, *p* = 0.00029, Figure 8(d)).

According to starBase, TargetScan, and miRDB, only PDL1 is targeted by hsa-miR-7-5p, while both PD1 and CTLA4 are not targeted gene of these 25 miRNAs.

### 3.6. GO and KEGG Analyses

**Figure 9:**
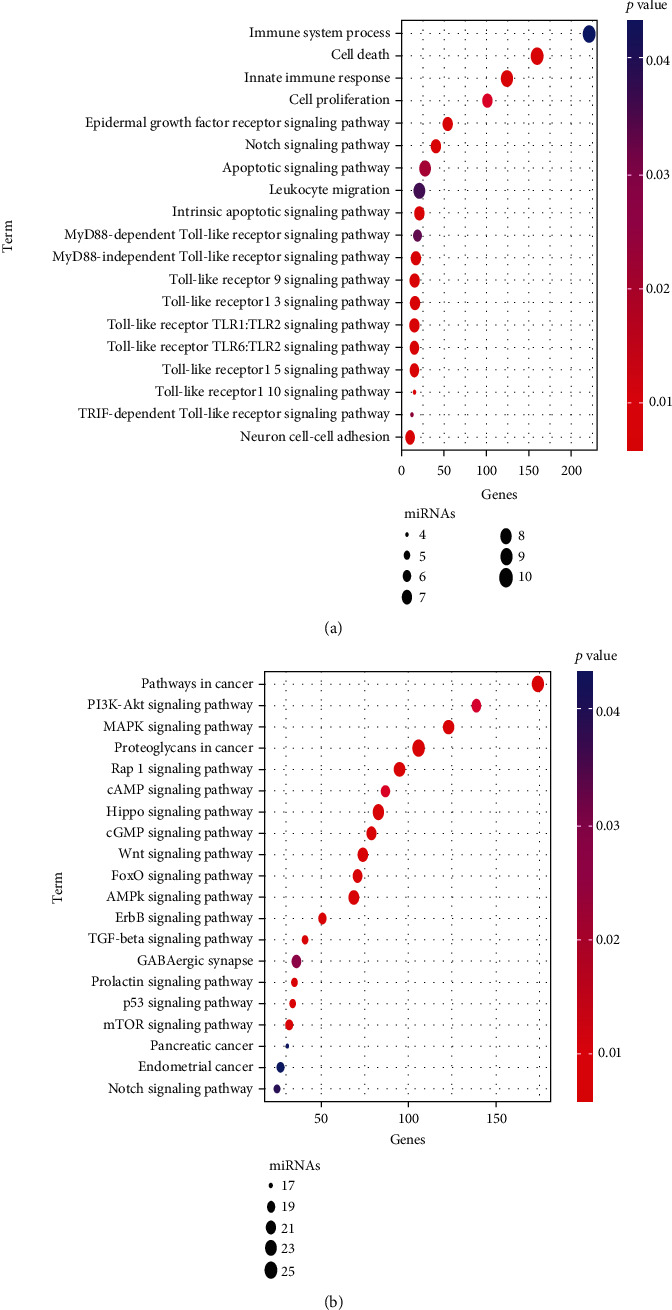
Functional enrichment analysis of the 25 miRNAs. (a) Significantly enriched immune-related biological process GO terms. (b) Significantly enriched cancer-related KEGG pathways.

The enrichment analysis was conducted to describe the biological function of the target of 25 miRNAs. It revealed enrichment of 301 Gene Ontology categories. The enrichment biological process is shown in Figure 9(a). Several immune-related biological processes were observed, including epidermal growth factor receptor signaling pathway, Toll-like receptor signaling pathway, leukocyte migration, and immune system process. A total of 58 KEGG pathways were enriched by the target genes (Figure 9(b)). Among these KEGG pathways, several cancer-related pathways were identified, including the Wnt signaling pathway, PI3K-AKT signaling pathway, P53 signaling pathway, and TGF-beta signaling pathway.

### 3.7. Association between miRNA-Based Classifier Index and Pathways

**Figure 10:**
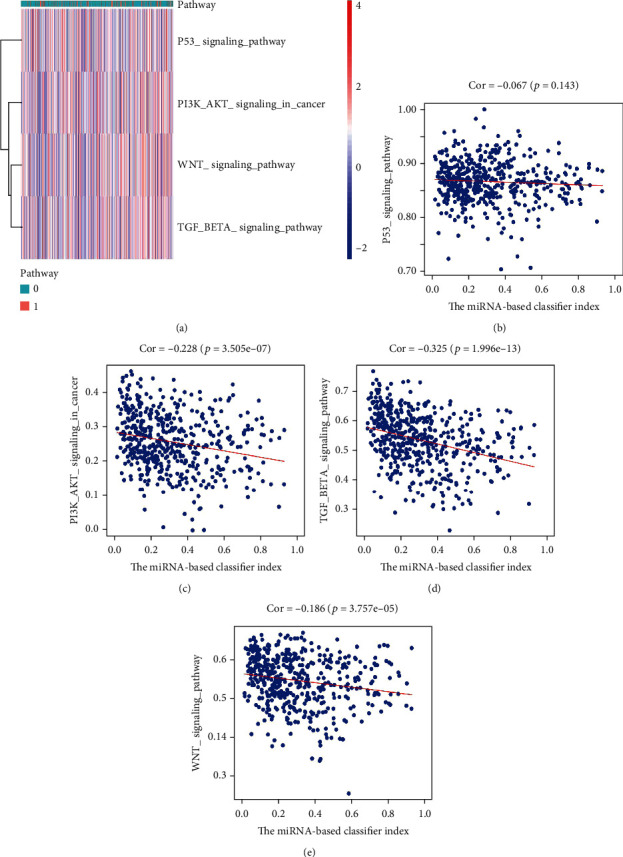
Association between miRNA-based classifier index and pathways. (a) Heatmap plot for the association between miRNA-based classifier index and pathways. (b)–(e) miRNA-based classifier index correlated with the P53 signaling pathway, PI3K-Akt signaling pathway, TGF-beta signaling pathway, and Wnt signaling pathway, respectively.

Single-sample gene set enrichment analysis (ssGSEA) was employed to impute the enrichment scores of interested pathways. The heatmap is exhibited in Figure 10(a). Meanwhile, we found that the miRNA-based classifier index was negatively correlated with the Wnt signaling pathway, PI3K-AKT signaling pathway, and TGF-beta signaling pathway Figure 10(b).

## 4. Discussion

Satisfactory results had been achieved in the immunotherapy for the treatment of aggressive or advanced cancers [32–34]. The progress of the tumor depends on the evasion of immune surveillance of cancer cells and the acquisition of effective immune response traits. HNSCC is considered to be an immunosuppressive disease. Its absolute lymphocyte count is lower than those found in a healthy population; it defects human leukocyte antigen (HLA), impairs the natural killer (NK) cell activity, and has poor antigen presentation function [35–37]. Impairment to tumor-infiltrating T lymphocytes has also been reported in HNSCC and other cancers, which has a great impact on the clinical outcome [38, 39]. Besides, inhibitory regulatory T cells (Tregs) are also associated with the tumor progression [40, 41]; for example, Treg secretes inhibitory cytokines, such as TGF-*β* and IL-10, and expresses cytotoxic T lymphocyte-associated protein 4 (CTLA4). Therefore, immunomodulation therapy to overcome the immunosuppressive signal of HNSCC patients has therapeutic prospects.

Although immunotherapy has shown satisfactory results for tumor therapy, only a small percentage of patients can benefit from it. Therefore, many studies have been designed to find predictive biomarkers of the immune response. At present, tumor mutational burden (TMB) is promising as another effective predictive biomarker for treatment with ICIs and independent of PDL1 expression [42, 43]. Multiple studies have shown that TMB can be effectively measured from liquid biopsy/blood, which may be an alternative method for biopsy [44]. However, TMB assessment by liquid biopsy must confront the problem that circulating DNA derived from tumor cells is usually only a small part of the DNA of noncirculating cells, and it does not reflect the true situation of tumor TMB [45].

Because of the key role of miRNAs in tumor-related immune responses, several studies reported that miRNA was involved in the development of HNSCC [46]. However, the association of miRNA expression patterns and TMB was not previously described in HNSCC. In the present work, the differentially expressed miRNAs between high TMB level and low TMB level samples are identified, and their expression patterns can distinguish high TMB level and low-level TMB samples. Using the LASSO model, a miRNA-based feature classifier was established in the training set and then verified in an independent test set. The accuracy of the 25-miRNA-based classifier is 0.850 in the training set, 0.810 in the test set, and 0.840 in the overall set. The miRNA-based feature classifier can predict TMB levels well.

In HNSCC, the interaction between programmed death receptor 1 (PD1) and its ligands PDL1 and PDL2 has been shown to downregulate T cell activation in human models [47]. Besides, PDL1 is often found overexpressed in >50-60% of HNSCC patients [48]. Targeted immune checkpoint receptors, including anti-CTLA4 and antiprogrammed death 1 (anti-PD1), provide further hope for patients to benefit from immune modulation. In the study, we found that the miRNA-based feature classifier is highly correlated with SNCA (PD1), CD274 (PDL1), and CTLA4. It has been widely speculated that high TMB levels leading to an increase in tumor neoantigens may trigger the immune system to attack the tumor. This study shows that multiple cancer-related miRNAs are differentially expressed among tumor samples with different TMB levels. The enrichment analysis for the miRNA-signature classifier suggested that the 25 miRNAs are involved in immune-related biological processes and cancer-related KEGG pathways, such as the Toll-like receptor signaling pathway, leukocyte migration, immune system process, Wnt signaling pathway, P53 signaling pathway, and TGF-beta signaling pathway.

In conclusion, HNSCC patients with different TMB levels have different miRNA expression patterns. A miRNA-based signature classifier was constructed and may be served as a biomarker to predict TMB levels in HNSCC. As our study results were derived from bioinformatic analysis, further clinical studies are needed to confirm these results.

## Figures and Tables

**Table 1:** Summary of basic clinical feature.

Covariates	Group	Total numbers (%)	Train numbers (%)	Test	*p* value
Age	≤65	324 (64.67%)	191 (63.46%)	133 (66.5%)	0.5466
	>65	177 (35.33%)	110 (36.54%)	67 (33.5%)	
Gender	Female	137 (27.35%)	81 (26.91%)	56 (28%)	0.8684
	Male	364 (72.65%)	220 (73.09%)	144 (72%)	
Grade	G1-2	360 (71.86%)	216 (71.76%)	144 (72%)	0.8565
	G3-4	122 (24.35%)	75 (24.92%)	47 (23.5%)	
	Unknown	19 (3.79%)	10 (3.32%)	9 (4.5%)	
Stage	Stage I-II	95 (18.96%)	60 (19.93%)	35 (17.5%)	0.5956
	Stage III-IV	338 (67.47%)	201 (66.78%)	137 (68.5%)	
	Unknown	68 (13.57%)	40 (13.29%)	28 (14%)	
T	T0-2	179 (35.73%)	108 (35.88%)	71 (35.5%)	1
	T3-4	266 (53.09%)	161 (53.49%)	105 (52.5%)	
	Unknown	56 (11.18%)	32 (10.63%)	24 (12%)	
M	M0	186 (37.13%)	114 (37.87%)	72 (36%)	1
	M1	1 (0.2%)	1 (0.33%)	0 (0%)	
	Unknown	314 (62.67%)	186 (61.79%)	128 (64%)	
N	N0	170 (33.93%)	114 (37.87%)	56 (28%)	0.0175
	N1-3	237 (47.31%)	130 (43.19%)	107 (53.5%)	
	Unknown	94 (18.76%)	57 (18.94%)	37 (18.5%)	

**Table 2:** Performance of 25-miRNA-based classifiers of tumor mutation burden in HNSCC.

Cohort	SE	SP	PPV	NPV	Accuracy	AUC
Train	0.4211	0.9369	0.7547	0.7782	0.7741	0.8222
Test	0.3607	0.8633	0.5366	0.7547	0.71	0.702
Total	0.3974	0.9072	0.6596	0.769	0.7485	0.774

## Data Availability

Data availability could be obtained from the TCGA website.

## Abbreviations

HNSCC: Head and neck squamous cell carcinoma
TMB: Tumor mutational burden
ICI: Immune checkpoint inhibitors
LASSO: The least absolute contraction and selection operator.
